# Treatment-seeking patients with binge-eating disorder in the Swedish national registers: clinical course and psychiatric comorbidity

**DOI:** 10.1186/s12888-016-0840-7

**Published:** 2016-05-26

**Authors:** Elisabeth Welch, Andreas Jangmo, Laura M. Thornton, Claes Norring, Yvonne von Hausswolff-Juhlin, Barry K. Herman, Manjiri Pawaskar, Henrik Larsson, Cynthia M. Bulik

**Affiliations:** Department of Medical Epidemiology and Biostatistics, Karolinska Institutet, Stockholm, Sweden; Department of Psychiatry, University of North Carolina at Chapel Hill, CB #7160, 101 Manning Drive, Chapel Hill, NC 27599-7160 USA; Stockholm Centre for Eating Disorders, Stockholm, Sweden; Department of Clinical Neuroscience, Karolinska Institutet, Stockholm, Sweden; Shire, Wayne, PA USA; Department of Nutrition, University of North Carolina at Chapel Hill, Chapel Hill, NC USA

**Keywords:** Binge-eating disorder, Comorbidity, Suicide, Eating disorders

## Abstract

**Background:**

We linked extensive longitudinal data from the Swedish national eating disorders quality registers and patient registers to explore clinical characteristics at diagnosis, diagnostic flux, psychiatric comorbidity, and suicide attempts in 850 individuals diagnosed with binge-eating disorder (BED).

**Method:**

Cases were all individuals who met criteria for BED in the quality registers (*N* = 850). We identified 10 controls for each identified case from the Multi-Generation Register matched on sex, and year, month, and county of birth. We evaluated characteristics of individuals with BED at evaluation and explored diagnostic flux across eating disorders presentations between evaluation and one-year follow-up. We applied conditional logistic regression models to assess the association of BED with each comorbid psychiatric disorder and with suicide attempts and explored whether risk for depression and suicide were differentially elevated in individuals with BED with or without comorbid obesity.

**Results:**

BED shows considerable diagnostic flux with other eating disorders over time, carries high psychiatric comorbidity burden with other eating disorders (OR 85.8; 95 % CI: 61.6, 119.4), major depressive disorder (OR 7.6; 95 % CI: 6.2, 9.3), bipolar disorder (OR 7.5; 95 % CI: 4.8, 11.9), anxiety disorders (OR 5.2; 95 % CI: 4.2, 6.4), and post-traumatic stress disorder (OR 4.3; 95 % CI: 3.2, 5.7) and is associated with elevated risk for suicide attempts (OR 1.8; 95 % CI: 1.2, 2.7). Depression and suicide attempt risk were elevated in individuals with BED with and without comorbid obesity.

**Conclusions:**

Considerable flux occurs across BED and other eating disorder diagnoses. The high psychiatric comorbidity and suicide risk underscore the severity and clinical complexity of BED.

## Background

Binge-eating disorder (BED), a recent addition to the DSM-5 [1], is marked by recurrent episodes of binge eating in the absence of recurrent inappropriate compensatory behaviors. Global DSM-IV lifetime prevalence estimates for BED are approximately 1.9 % in adults [2]. An internet-based study of 22,397 adults [Validate Attitudes and Lifestyle Issues in Depression ADHD and Troubles with Eating (VALIDATE)], reported lifetime prevalence estimates of DSM-5 BED projected to the US population to be 2.03 %, (1.41 % for men and 2.61 % for women) [3]. Age at onset is typically late teens to early twenties [2, 4]. The illness can be protracted and can wax and wane [2, 4–8]. We explored fundamental features of BED including clinical characteristics, diagnostic flux, and psychiatric comorbidities, using rich, extensive, Swedish population register data.

Diagnostic flux (i.e., migration from one eating disorders presentation to another) commonly occurs between anorexia nervosa (AN) and bulimia nervosa (BN) [5, 6, 8–11]; however, less is known regarding flux in BED. Crossover between BED and BN [5, 6] and between BED and eating disorder not otherwise specified (EDNOS) [5] has been reported; however, large-scale, longitudinal, population-based explorations are lacking.

Psychiatric comorbidity burden is considerable in individuals with BED with the most common co-occurring conditions being mood [12–20], anxiety [13–20] and substance use disorders [12, 14, 16, 17, 20]. Using national patient records, we are able to broaden the inquiry to include more psychiatric disorders. An important question addressed in the literature is the extent to which morbidity and comorbidity in BED is attributable to the presence of obesity. Hudson et al. [4] have shown that risk for components of metabolic syndrome are elevated in BED independent of the effects of BMI. We further explored the extent to which observed psychiatric comorbidity in BED was attributable to obesity.

Register-based studies that include longitudinal follow-up and that capture all clinical contacts in a nation’s health care system allow for intensive characterization of a disorder. By linking rich longitudinal data from the Swedish national eating disorders quality registers and national patient registers, we explored clinical characteristics at evaluation, diagnostic flux, and psychiatric comorbidity in all individuals presenting for evaluation for BED across the country.

## Methods

### Procedure

Data were extracted in 2009 from Swedish population registers, which can be linked via a unique personal identification number assigned to all Swedish residents. Specifically, we linked a) the eating disorders national quality registers, Riksät-National Quality Register for Eating Disorders Treatment [21] and Stepwise [22] which first entered patient information in 1999 and 2005; b) the NPR [23], which provides national coverage of all Swedish public and private hospital inpatient admissions beginning in 1973 and outpatient specialist care beginning in 2001; c) the Multi-Generation Register [24], which can be used to determine biological and adoptive relationships for all individuals living in Sweden since 1933; d) the Migration Register [25]; and e) the Cause of Death Register [26], which contains the date and principal and contributing cause(s) of deaths since 1958. Details about the registers are provided elsewhere [27]. Height and weight were assessed at evaluation.

Informed consent is not required for inclusion in the Swedish registers, except for Stepwise in which research participation is elective [28]. The University of North Carolina Biomedical Institutional Review Board and the Regional Ethics Committee of Karolinska Institutet both approved this study.

### Sample

Cases were defined as any individual with a BED diagnosis in Riksät [21] or Stepwise [22] at any evaluation point. Briefly, Riksät is an Internet-based register for treatment of eating disorders that includes eating disorders-specific information on approximately 8,600 patients. Follow-up assessments occur on a yearly basis as long as the patient is in treatment. Eating disorder diagnoses are determined at each assessment using standard clinical assessments and BED is diagnosed specifically and not subsumed under EDNOS.

All patients included in Stepwise are in Riksät, but the reverse is not true. Stepwise contains more detailed information on clinical presentation, course, outcome, and psychopathology. Once intent to treat is established (usually within one to three weeks), eating disorders are assessed by clinicians using structured diagnostic interviews assessing DSM-IV criteria [29]. Stepwise includes initial data on approximately 2,700 eating disorders patients. Twelve-month follow-up data exist for ~69 % of the patients registered in Riksät or Stepwise. Inclusion criteria for Riksät and Stepwise are: a) medical or self-referral to a participating treatment unit, b) a diagnosed eating disorder, and c) intent to treat. Between them, Riksät and Stepwise cover almost all specialized eating disorder units in Sweden (in 2009 approximately 90 %), and many non-specialized general psychiatric units.

We used the Multi-Generation Register to identify 10 controls for each case. Controls were matched to cases based on sex, year and month of birth, and county of birth. Cases born outside of Sweden were matched on immigration status and time of migration (controls could not immigrate later than their respective cases), regardless of origin. Controls had to be alive and resident in Sweden for an equal period of time: from birth or immigration up until the time of diagnosis for their respective case. They also could not have received a BED diagnosis in Riksät or Stepwise, but could have had another eating disorder (detected in 1.0 % of controls).

### Psychiatric diagnoses

Diagnostic information for psychiatric disorders and suicide (see Table 1) for all cases and controls were obtained from the NPR. Diagnoses were based on WHO *International Classification of Diseases, Ninth Revision* (ICD-9: years 1987–1996) or *International Classification of Diseases, Tenth Revision* (ICD–10: years 1997–present).

**Table 1:** ICD-9 and ICD-10 codes for other psychiatric diagnoses from the National Patient Register

Diagnosis	ICD9	ICD10
Schizophrenia	295A–295E, 295G, 295W, 295X	F20, F20.0–F20.6, F20.8 F20.9
Schizoaffective disorder	295H	F25, F25.0–F25.2, F25.8, F25.9, F23.1, F23.2
Bipolar disorder	296A, 296C–296H, 296W, 296X	F30, F30.1, F30.2, F30.8, F30.9, F31, F31.0–F31.9, F34.0
Major depressive disorder	296B, 300E, 311	F32, F32.0–F32.3, F32.8, F32.9, F33, F33.0–F33.4, F33.8, F33.9, F34.1, F34.8, F34.9, F38, F38.0, F38.1, F38.8, F39
Any anxiety disorder^a^	300A, 300C	F40, F40.0–F40.2, F40.8, F40.9, F41, F41.0–F41.3, F41.8, F41.9
Obsessive-compulsive disorder	300D	F42, F42.0–F42.2, F42.8, F42.9
Post-traumatic stress disorder	308, 309A, 309B, 309W, 309X	F43, F43.0–F43.2, F43.8, F43.9
Attention deficit hyperactivity disorder	314 J, 314W, 314X	F90, F90.0, F90.1, F90.8
Autism	299A	F84.0, F84.1, F84.5
Alcohol use disorder	303, 303A, 303X, 305A	F10, F10.0–F10.9
Drug use disorder	304, 304A–304H, 304W, 304X, 305X	F11, F11.0–F11.9, F12, F12.0–F12.9, F13, F13.0–F13.9, F14, F14.0–F14.9, F15, F15.0–F15.9, F16, F16.0–F16.9, F18, F18.0–F18.9, F19, F19.0–F19.9
Anorexia nervosa	307B	F50.0, F50.1
Bulimia nervosa	–	F50.2, F50.3
Any eating disorder	307B, 307F	F50.0–F50.5, F50.8, F50.9
Suicide attempts/completions	E95A–E95H, E95W, E95X	X60–X84

^a^Any anxiety disorder does not include obsessive-compulsive disorder or post-traumatic stress disorder

### Data analysis

Analyses were conducted using SAS version 9.4 [30]. We first evaluated characteristics of individuals with BED at the initial visit and explored diagnostic flux from the initial visit to the first follow-up visit in individuals who were diagnosed with BED at either of these occasions. We report diagnosis at evaluation and diagnosis at one-year follow-up. Second, conditional logistic regression models were applied to assess the association of BED status (case/control) with each comorbid psychiatric disorder. Third, given diagnostic flux, sensitivity analyses evaluating the association between BED status and the psychiatric disorders were conducted excluding cases (and their respective controls) who had an additional eating disorder diagnosis (other than BED) in Riksät or Stepwise or who had a diagnosis of AN or BN in the NPR. These sensitivity analyses evaluated the association of BED and other psychiatric disorders free from confounding of other detected eating disorders. Sex differences in all models were explored. All tests were two-tailed. Given reported associations between obesity and depression and suicidality [31], we explored whether obesity (BMI ≥ 30 kg/m^2^) was associated with increased risk of depression and attempted suicide in individuals with BED while controlling for sex, and county and year of birth.

## Results

### Sample description

A total of 850 individuals, (4.6 % male), were identified with a diagnosis of BED. Table 2 presents the characteristics of the BED sample at evaluation. The age range for first BED diagnosis was 14–72 years (mode = 22 years). Men were significantly older, had significantly higher BMIs, and were more likely to be immigrants than women. The majority (67 %) attended a follow-up registration for Riksät/Stepwise, on average, 1.5 years after their initial visit.

**Table 2:** Characteristics of BED cases at initial visit to clinic and results comparing men with women

	All	Women	Men	Statistical test: value (*p*-value)
Total Sample: *N* (%)	850 (100.0)	811 (95.4)	39 (4.6)	
Received BED diagnoses only: *N* (%)	607 (71.4)	575 (70.9)	32 (82.1)	*χ* ^2^: 2.27 (.13)
Age at first visit: mode	22	22	25	t: 2.47 (.01)*
BMI: mean (std)	29.8 (8.4)	29.7 (8.3)	32.7 (9.4)	t: 2.20 (.03)
At least one follow-up visit: *N* (%)	571 (67.2)	546 (67.3)	25 (64.1)	*χ* ^2^: 0.18 (.68)
Days to first follow-up visit: mean (std)	548.4 (411.6)	546.6 (413.8)	586.8 (365.0)	t: 1.40 (.16)*
Immigrated: *N* (%)	73 (8.6)	66 (8.1)	7 (17.9)	*χ* ^2^: 4.56 (.03)

*Wilcoxon rank sum test

**Fig. 1:**
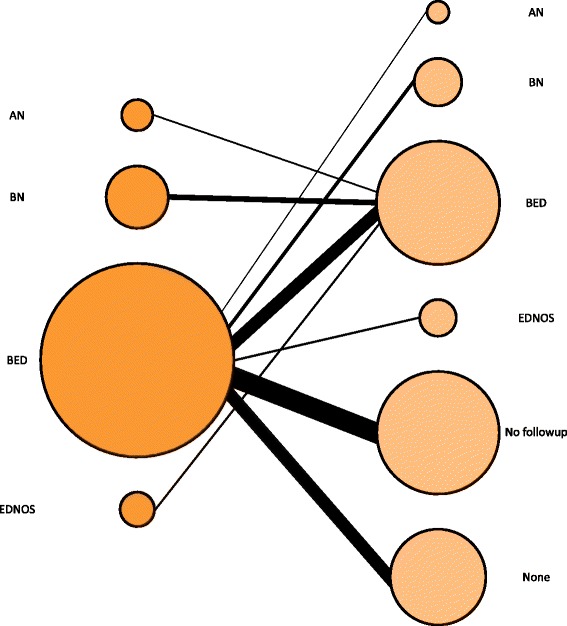
Diagnostic flux in individuals with BED. The sample included only those individuals who received a diagnosis of BED at the initial visit or at first follow-up visit. (Note: AN = anorexia nervosa, BN = bulimia nervosa, BED = binge-eating disorder, EDNOS = eating disorder not otherwise specified)

Out of the 850 individuals, 807 (94.9 %) received their BED diagnosis either at evaluation or one year follow-up. The remaining 43 individuals were first diagnosed with BED at a later follow-up. Figure 1 depicts the changes in eating disorder diagnoses between the initial visit and one year follow-up visit for these 807 individuals. Of the 695 individuals diagnosed with BED at evaluation, 279 (40.1 %) did not have a follow-up visit. However, 170 (24.5 %) received a second BED diagnosis at follow-up and 170 (24.5 %) received no eating disorder diagnosis at follow-up. In addition, 112 individuals were diagnosed with AN, BN, or EDNOS at evaluation, but received a diagnosis of BED at one-year follow-up.

Associations between BED and each psychiatric disorder in the full sample are presented in Table 3. BED was significantly associated with all psychiatric disorders evaluated except schizophrenia, schizoaffective disorder, and autism, where power was too low to draw meaningful conclusions. The strongest associations were with other eating disorders, particularly BN, and the composite “any eating disorder” category. Of the other disorders, major depressive disorder (MDD) and bipolar disorder had the strongest associations with BED followed by any anxiety disorder and post-traumatic stress disorder (PTSD). Suicide risk, even after controlling for MDD, was significantly elevated in individuals with BED. Sex differences could not be evaluated for 11 disorders because of small cell sizes, and no sex differences were observed for any anxiety disorder, MDD, BN, and any eating disorder.

**Table 3:** *N* (%) of individuals with each psychiatric disorder by BED group (case/control) and results of conditional logistic regressions [OR (95 % CI)] evaluating the association of BED with each psychiatric disorder

	BED (Cases) *N* = 850	Controls *N* = 8,500	
	*N* (%)	*N* (%)	OR (95 % CI)
Schizophrenia	1 (0.1)	17 (0.2)	0.6 (0.1, 4.7)
Schizoaffective disorder	1 (0.1)	10 (0.1)	1.1 (0.1, 8.8)
Bipolar disorder	35 (4.2)	49 (0.6)	7.5 (4.8, 11.9)
Major depressive disorder	203 (23.9)	337 (4.0)	7.6 (6.2, 9.3)
Any anxiety disorder^a^	146 (17.2)	315 (3.7)	5.2 (4.2, 6.4)
Obsessive-compulsive disorder	10 (1.2)	37 (0.4)	2.6 (1.2, 5.5)
Post-traumatic stress disorder	80 (9.4)	209 (2.5)	4.3 (3.2, 5.7)
Attention deficit hyperactivity disorder	14 (1.7)	51 (0.6)	3.1 (1.7, 5.6)
Autism	2 (0.2)	27 (0.3)	0.4 (0.1, 3.0)
Alcohol use disorder	27 (3.2)	152 (1.8)	1.7 (1.1, 2.7)
Drug use disorder	18 (2.1)	94 (1.1)	1.9 (1.1, 3.2)
Anorexia nervosa	40 (4.7)	37 (0.4)	10.6 (6.7, 16.8)
Bulimia nervosa	96 (11.3)	11 (0.1)	99.9 (50.4, 198.3)
Any eating disorder	375 (44.1)	83 (1.0)	85.8 (61.6, 119.4)
Suicide attempts/completions	49 (5.8)	139 (1.6)	3.6 (2.5, 5.0)
Suicide attempts/completions^b^	49 (5.8)	139 (1.6)	1.8 (1.2, 2.7)

^a^Any anxiety disorder does not include obsessive-compulsive disorder or post-traumatic stress disorder

^b^After controlling for the presence of major depressive disorder

Results from the sensitivity analyses, where cases with other lifetime eating disorder diagnoses and their controls were removed, are presented in Table 4. The associations between BED and comorbid disorders remained significant, with the exception of drug use disorder and suicide. In addition, we compared comorbidity profiles in individuals who returned for follow-up versus those who did not. The only difference was a greater likelihood of reporting alcohol use disorders in those who did not return (data not shown).

**Table 4:** *N* (%) of individuals with each psychiatric disorder by BED group (case/control), excluding cases who received other eating disorder diagnoses than BED and their controls, and results of conditional logistic regressions [OR (95 % CI)] evaluating the association of BED with each psychiatric disorder

	BED (Cases) *N* = 549	Controls *N* = 5,490	
	*N* (%)	*N* (%)	OR (95 % CI)
Schizophrenia	1 (0.2)	16 (0.3)	0.7 (0.1, 5.0)
Schizoaffective disorder	1 (0.2)	6 (0.1)	2.0 (0.2, 17.1)
Bipolar disorder	25 (4.6)	35 (0.6)	7.6 (4.4, 13.0)
Major depressive disorder	130 (23.7)	227 (4.1)	7.1 (5.6, 9.2)
Any anxiety disorder^a^	93 (16.9)	205 (3.7)	5.0 (3.8, 6.5)
Obsessive-compulsive disorder	5 (0.9)	20 (0.4)	2.9 (1.0, 7.9)
Post-traumatic stress disorder	56 (10.2)	134 (2.4)	4.6 (3.3, 6.5)
Attention deficit hyperactivity disorder	11 (2.0)	35 (0.6)	3.5 (1.8, 7.1)
Autism	1 (0.2)	16 (0.3)	0.5 (0.0, 2.4)
Alcohol use disorder	18 (3.3)	95 (1.7)	1.9 (1.1, 3.2)
Drug use disorder	9 (1.6)	67 (1.2)	1.4 (0.7, 2.8)
Suicide attempts/completions	25 (4.6)	88 (1.6)	2.8 (1.8, 4.5)
Suicide attempts/completions^b^	25 (4.6)	88 (1.6)	1.5 (0.9, 2.7)

^a^Any anxiety disorder does not include obsessive-compulsive disorder or post-traumatic stress disorder

^b^Controlling for major depressive disorder

Finally, the OR for MDD was significantly elevated in individuals with BED with comorbid obesity, ONLY when including individuals who also had other lifetime eating disorders. When confining the analyses to individuals who ONLY had a BED diagnosis, there was no significant elevation in MDD associated with comorbid obesity. The risk of suicide attempts in individuals with BED with or without comorbid obesity did not differ significantly.

## Discussion

Using the extensive register data available for the entire country of Sweden, we were afforded the unique opportunity to explore clinical course and psychiatric comorbidity of all individuals seeking treatment for BED, yielding a sample of 850 individuals. Briefly, BED shows considerable diagnostic fluidity with other eating disorders, carries high psychiatric comorbidity burden, and is associated with elevated suicide risk.

The reported age range at first diagnosis was broad (from 14–72), encouraging vigilance for BED across the age spectrum. Less than 5 % of the sample was male, which could reflect a lower prevalence of BED in males in Sweden (contrast the projected lifetime prevalence of 1.41 % projected for men in the US [3]), or, that males with BED are less likely to seek assistance for their disordered eating than females [32]. A recent evidence based review reported that across 48 reviewed randomized controlled trials, the percentage of females participating ranged from 67 to 100 % [33] suggesting that relative to epidemiological prevalence estimates, females do indeed appear to be more likely to seek treatment. Therefore, treatment seeking (not just in Sweden) may be less common in males with BED, underscoring the importance of psychoeducation that reaches males as well as clinicians and other positioned to detect BED in males.

Fortuitously, 67 % of evaluated individuals returned for a follow-up visit allowing us to capture detailed clinical course and symptom progression, although, by the nature of the quality registers, we are unable to tether those changes to specific interventions. One quarter of the returnees received a second diagnosis of BED at follow-up and one quarter received no eating disorder diagnosis reflecting remission of symptoms. Sixteen percent of those returning for follow-up received a different eating disorder diagnosis—most commonly BN or EDNOS and occasionally AN. This novel observation is of clinical importance in encouraging clinicians to be mindful of signs of transitioning to other eating disorder presentations. Those who transitioned to EDNOS may have been on a recovery trajectory and the change in diagnosis could reflect a reduction in total symptomatology (e.g., binge frequency) reflecting a subthreshold presentation. Such changes will presumably be rectified with the inclusion of remission specifiers in DSM-5. Transitions to BN and AN do not reflect a remission trajectory, but rather reflect the emergence of recurrent inappropriate compensatory behaviors (in BN) or significant weight loss and/or restricting behaviors (in AN), both concerning clinical developments. Of note, flux was bidirectional, with patients also transitioning to BED from AN, BN, or EDNOS. Given that our design only included at maximum two visits, and also reported on individuals who only made one visit to a clinic, although we can describe considerable diagnostic flux, we are not well positioned to comment authoritatively on the long-term stability of BED as a diagnostic entity.

We confirm and extend previous reports of comorbidity in BED [12–20]. The greatest lifetime comorbidity burden was with the other eating disorders with the large odds ratio dramatically highlighting the diagnostic fluidity of these illnesses and encouraging further investigation into the mechanisms underlying diagnostic instability. Genetic, neurobiological, and environmental influences should be explored and risk algorithms developed to aid in the prediction of clinical course.

Of the other psychiatric disorders, the risk of MDD and bipolar disorder were most elevated, followed by anxiety disorders and PTSD. Only risk for schizophrenia, schizoaffective disorder, and autism spectrum disorders were not elevated. Elevated risk for depression was not associated with comorbid obesity in the “pure BED” (i.e., no other eating disorder diagnoses) sample. Thus it should not be assumed that normal weight individuals with BED are at any less risk for depression. These observations underscore the substantial comorbidity burden carried by individuals with BED and highlight the clinical importance of assessing and addressing the full array of presenting problems facing these patients. Treatment research should address the extent to which interventions require tailoring based on comorbidity profile.

Of particular clinical concern is the elevated risk for suicide in individuals with BED. Even when controlling for the presence of MDD, those with BED were at significantly increased risk for suicide attempts. Our sensitivity analyses revealed that suicide risk was elevated regardless of the presence of comorbid obesity. These results extend previous reports [34] and underscore the seriousness of BED as a psychiatric illness.

Our observations of elevated suicide risk in BED are important clinically; however, alternative designs are necessary to explicate the mechanism of the association [35]. One possibility is that a factor such as impulsivity could represent a genetically influenced intermediate phenotype behind both BED [36, 37] and some, but not all forms of suicidal behavior [38–40]. Alternatively or complementarily, environmental risk factors (e.g., sexual and physical abuse, bullying or being bullied) could represent important shared risk factors for BED [41] and suicidal behavior [42, 43].

The comparison of individuals with pure BED versus those with lifetime histories of other eating disorders revealed similarly high comorbidity burden, with the exception of drug use and suicide. This observation suggests that suicide risk in BED may be particularly concerning in those who have transitioned to or from other eating disorders during the trajectory of their illness.

Our study has notable limitations. First, we only included individuals who presented for an evaluation and received a diagnosis of BED. Untreated individuals in the community may present with different patterns of clinical course, comorbidity, and suicide risk. Second, we did not screen for eating disorders in controls. As such, our analyses represent conservative comparisons and the actual associations may be stronger. This approach was taken to guard against inflated estimates caused by artificially “clean” control samples. Third, as personality disorders tend to be less reliably included in patient registers, we were unable to explore comorbidity between BED and personality disorders. Fourth, assuming variable and incalculable lags between symptom onset and diagnosis across disorders, reports on the temporality of diagnoses could reflect either true order of onset of the disorders or differential latency to treatment seeking. Fifth, 33 % of patients did not return for follow-up. With the exception of alcohol use disorders, their comorbidity profiles were no more severe. Those who did not return may not have been recommended for further treatment at a specialty service or could have opted out of treatment.

Balancing these limitations are several methodological strengths including the large sample size, standardized evaluation by trained clinicians and the longitudinal follow up afforded by the Swedish eating disorders quality registers, national healthcare records, and detailed register-based patient psychiatric and other medical history.

## Conclusion

In conclusion, rich data from the Swedish registers on 850 individuals with BED reveal considerable diagnostic fluidity across eating disorders presentations, high comorbidity burden, and elevated suicide risk. Clinicians should remain vigilant for a broad array of comorbid conditions, for the emergence of inappropriate compensatory behaviors or restrictive eating, and for suicidal ideation in patients with BED.

## Abbreviations

AN: anorexia nervosa
BED: binge-eating disorder
BMI: body mass index
BN: bulimia nervosa
CI: confidence interval
DSM-5: Diagnostic and Statistical Manual of Mental Disorders, fifth edition
EDNOS: eating disorder not otherwise specified
ICD-9: International Classification of Diseases, ninth revision
IDC-10: International Classification of Diseases, tenth revision
MDD: major depressive disorder
NPR: National Patient Registry
OR: odds ratio
PTSD: post-traumatic stress disorder
VALIDATE: Validate Attitudes and Lifestyle Issues in Depression ADHD and Troubles with Eating
